# MiR-27a-3p promotes the osteogenic differentiation by activating CRY2/ERK1/2 axis

**DOI:** 10.1186/s10020-021-00303-5

**Published:** 2021-04-26

**Authors:** Li-Rong Ren, Ru-Bin Yao, Shi-Yong Wang, Xiang-Dong Gong, Ji-Tao Xu, Kai-Shun Yang

**Affiliations:** Department of Spine Surgery, The First Affiliated Hospital of Dali University, No.32, Jiashibo Avenue, Dali, 671000 Yunnan Province People’s Republic of China

**Keywords:** Osteoporosis, Osteogenic differentiation, ERK1/2 pathway, miR-27a-3p

## Abstract

**Background:**

Osteoporosis seriously disturbs the life of people. Meanwhile, inhibition or weakening of osteogenic differentiation is one of the important factors in the pathogenesis of osteoporosis. It was reported that miR-27a-3p reduced the symptoms of osteoporosis. However, the mechanism by which miR-27a-3p in osteogenic differentiation remains largely unknown.

**Methods:**

To induce the osteogenic differentiation in MC3T3-E1 cells, cells were treated with osteogenic induction medium (OIM). RT-qPCR was used to evaluate the mRNA expression of miR-27a-3p and CRY2 in cells. The protein levels of CRY2, Runt-related transcription factor 2 (Runx2), osteopontin (OPN), osteocalcin (OCN) and the phosphorylation level of extracellular regulated protein kinases (ERK) 1/2 in MC3T3-E1 cells were evaluated by western blotting. Meanwhile, calcium nodules and ALP activity were tested by alizarin red staining and ALP kit, respectively. Luciferase reporter gene assay was used to analyze the correlation between CRY2 and miR-27a-3p.

**Results:**

The expression of miR-27a-3p and the phosphorylation level of ERK1/2 were increased by OIM in MC3T3-E1 cells, while CRY2 expression was decreased. In addition, OIM-induced increase of calcified nodules, ALP content and osteogenesis-related protein expression was significantly reversed by downregulation of miR-27a-3p and overexpression of CRY2. In addition, miR-27a-3p directly targeted CRY2 and negatively regulated CRY2. Meanwhile, the inhibitory effect of miR-27a-3p inhibitor on osteogenic differentiation was reversed by knockdown of CRY2 or using honokiol (ERK1/2 signal activator). Furthermore, miR-27a-3p significantly inhibited the apoptosis of MC3T3-E1 cells treated by OIM. Taken together, miR-27a-3p/CRY2/ERK axis plays an important role in osteoblast differentiation.

**Conclusions:**

MiR-27a-3p promoted osteoblast differentiation via mediation of CRY2/ERK1/2 axis. Thereby, miR-27a-3p might serve as a new target for the treatment of osteoporosis.

## Background

Osteoporosis is a common bone disease in aging people, and the imbalance of bone formation and bone resorption are the major causes of the progression of osteoporosis (Katri et al. 2019; Rachner et al. 2011). The risk of fractures in the limbs and trunk were greatly increased due to the development of osteoporosis, which could result in 30–50 % mortality (Hirsch 2018). The steady state of bones was mainly maintained by osteoblasts and osteoclasts. Osteoblasts mainly originates from bone marrow mesenchymal stem/stromal cells, which played key roles in bone mass regulation (Teitelbaum 2000). Osteoclasts were large multinucleated cells derived from the mononuclear macrophages, which could absorb bone and promote bone renewal (Boyle et al. 2003). The abnormal differentiation of osteoblasts and osteoclasts resulted in the loss of bone mass and bone structure strength, and they ultimately led to the occurrence of osteoporosis (Boot et al. 2016). Therefore, screening and identifying drugs that inhibit osteoclasts or promote osteoblast differentiation is essential for developing new treatment methods against osteoporosis.

Autophagy stands for an ability of cells to prevent their own death, and apoptosis was the common cellular process (Ling et al. 2021). In addition, autophagy could also protect cells against apoptosis (Bai et al. 2021). Clock genes [such as cryptochromes (Cry1 and Cry2) and cycles (Per, Per2 and Per3)] are known to be timing system formed in the human body which effectively modulate the autophagy in cells (Partch et al. 2014). Meanwhile, silencing of CRY2 accelerated the acetylation of histone 3 by activating the CLOCK/BMAL1/P300 signaling pathway, and then promoted the osteogenic bone formation and cell autophagy through promoting a transcription complex with Runt-related transcription factor 2 (Runx2) (Tang et al. 2020). Additionally, previous studies showed that regulating the intracellular activity of CRY1 could be used to regulate osteogenesis and fat formation (Kushibiki and Awazu 2008), suggesting the possible relationship between CRY and osteoporosis. On the other hand, cells will switch to an autophagy pathway when apoptosis is inhibited (Yu et al. 2021). It was previously reported that autophagy and apoptosis were closely associated with the differentiation of osteoblasts (Dai et al. 2021). Moreover, mitogen-activated protein kinase (MAPK) could regulate cell proliferation, apoptosis and differentiation (Johnson and Lapadat 2002). Extracellular regulated protein kinases (ERK) were highly expressed in osteoblasts, including ERK1 (MAPK3) and ERK2 (MAPK1) type (Kim et al. 2019). Honokiol (2-(4-hydroxy-3-prop-2-enyl-phenyl)-4-prop-2-enylphenol) is a small-molecule polyphenol, which is the most important active pharmaceutical ingredient of Houpo (Yeh et al. 2016). Furthermore, honokiol could induce the activation of ERK signaling pathway (Yeh et al. 2016). Honokiol and p-ERK promoted osteoblast differentiation (Boot et al. 2016; Yeh et al. 2016). Multiple reports indicated that ERK1/2 pathway could promote osteoblast differentiation and bone formation (Kim et al. 2019). Besides, puerarin and astragalin promoted osteogenic differentiation in MC3T3 and MG-63 cells by activating ERK1/2 to prevent osteoporosis (Liu et al. 2019; Wang et al. 2013). Furthermore, CRY2 knockdown increased the phosphorylation of ERK1/2 by inducing cell cycle progression in tumor cells (Yu et al. 2018). However, the relationship between these two ingredients in osteoblast differentiation has not been reported.

MicroRNAs (miRNAs) play crucial regulatory roles in the process of bone remodeling, especially in the regulation of bone structure that balances bone formation, bone resorption, and the development of osteoporosis (Ge et al. 2017). Meanwhile, a study revealed that miR-27a-3p was involved in various diseases. For instance, BMSC-derived extracellular vesicles carrying miR-22-3p could promote osteogenic differentiation and bone formation by mediation of the FTO (Zhang et al. 2020). Fu et al. found that miR-27a-3p induced osteogenic differentiation in hMSC via activation of osteogenic genes (Fu et al. 2019). However, the detailed association between miR-27a-3p and ERK1/2 pathway in osteogenic differentiation needs to be further explored. Therefore, the application of miR-27a-3p in novel osteoporosis therapy is worthy of attention, and the further research is needed. Based on these backgrounds, the aim of this study was to investigate the function of miR-27a-3p in pre-osteoblast differentiation. In addition, the role ERK1/2 signal transduction in miR-27a-3p-mediated osteogenesis would be investigated. We hoped this research would provide new ideas for exploring the new therapeutic strategy against osteoporosis.

## Methods

### Cell culture and differentiation

The pre-osteoblast MC3T3-E1 was obtained from the cell bank of the Chinese Academy of Sciences (Shanghai, China). Cells were cultured in Dulbecco’s modified Eagle’s medium (DMEM) containing 10 % fetal bovine serum (FBS; Gibco, CA, USA). Osteoblastic differentiation of MC3T3-E1 cells was induced by adding a mixture of 10 % FBS, L-ascorbic acid (10 mM), dexamethasone (10^− 8^ mol/L) and β-glycerophosphate (10 mM) (Takara Bio, Shiga, Japan) every 2 days. Meanwhile, honokiol was obtained from MedChemExpress (MCE; 10 nM, Shanghai, China). In addition, it was dissolved in 10 mL DMSO and diluted with purified water (1:100).

### Mineralization analysis

The degree of osteogenic differentiation of MC3T3-E1 cells (ODM) was evaluated by cell matrix mineralization after staining with Alizarin Red S (Sigma-Aldrich, MA, USA). After treatment with MK430, cells were washed twice with PBS and fixed with ice-cold 70 % ethanol for 30 min at room temperature. After washing with PBS for three times, cells were stained with Alizarin Red S for 30 min at room temperature. Finally, the microscope (Olympus Corporation, Japan) was used to observe the calcium deposit at 30× magnification, and ten random fields were selected using Image-Pro Plus software (Media Cybernetics) to quantify matrix mineralization.

### ALP enzyme assay

According to the product manual, ALP kit (Excellbio, Shanghai, China) was used to investigate the level of ALP in MC3T3-E1 cells. 100 µL of serially diluted standard samples and MC3T3-E1 serially diluted lysed samples were added to microplates, respectively. After 2 h of incubation at 37 °C, cells were stained with 100 µL ALP antibody at 37 °C for 1 h. Then, cells were incubated with 100 µL secondary antibody at 37 °C for 30 min. Finally, the specific binding optical density of each well was determined at 450 nm using a microplate reader (Bio-Rad, Hercules, CA, USA) after inoculated with 100 µL substrate in the dark for 15 min.

### Quantitative real‐time PCR

According to the manufacturer’s instructions, total RNA was extracted from cells using rapid extraction kit (BioTeke, Beijing, China) according to the manufacturer’s instruction. 2 µg RNA was reversed transcribed into cDNA by TaqMan Micro-RNA Reverse Transcription Kit (Applied Biosystems, Foster, CA). Real-time quantitative PCR was performed using SYBR Green I Master Mix (Solarbio, Beijing, China) on and Exicycler 96 quantitative PCR analyzer (Bioneer, Daejeon, South Korea). The reaction procedure was as follows: 95 °C, 3 min, 95 °C, 30 s, 58 °C, 30 s for 40 cycles. The expression of miR-27a-3p and CRY2 was calculated normalized to U6 and GAPDH with 2^−△△Ct^ method. The primers used were as follows: miR-27a-3p, 5′-GCGCATTCACAGTGGCTAAG-3′ (forward) and 5′-GTCGTATCCAGTGCAGGGTCCGAGGTATTCGCACTGGATACGACGCGGAA-3′ (reverse); CRY2, 5′-GGGACTCTGTCTATTGGCATCTG-3′ (forward) and 5′-GTCACTCTAGCCCGCTTGGT-3′ (reverse); U6, 5′-CTCGCTTCGGCAGCACA-3′ (forward) and 5′-AACGCTTCACGAATTTGCGT-3′ (reverse); GAPDH, 5′-AGCCCAAGATGCCCTTCAGT-3′ (forward) and 5′-CCGTGTTCCTACCCCCAATG − 3′ (reverse).

### Cell transfection

Oligonucleotides (negative controls, miR-27a-3p inhibitor, miR-27a-3p mimics and CRY2 overexpression plasmids) were purchased from GenePharma (Shanghai, China). Furthermore, for CRY2 knockdown, the short hairpin RNA (shRNA) specific for CRY2 (sh-CRY2) or negative control shRNA (sh-NC) was obtain from GenePharma (Shanghai, China). MC3T3-E1 cells were transfected with miR-NC, miR-27a-3p mimics, miR-27a-3p inhibitor, pcDNA-CRY2 vector, sh-CRY2 and sh-NC using Lipofectamine 2000 (Invitrogen, Thermo Fisher Scientific) according to the manufacturer’s instructions. Finally, all transfected cells were collected for experiments.

### Western blotting

Cells were harvested and lysed in RIPA lysis buffer (Biyuntian Biotech Co., Ltd., Beijing, China). Then, the BCA analysis kit (Bio-Rad) was used to quantify the protein concentration. The proteins (25 µg per lane) were separated by 10 % SDS-PAGE and then transferred onto polyvinylidene fluoride membrane (PVDF) (EMD Millipore, Billerica, MA, USA). The PVDF membrane was blocked in 5 % skimmed milk powder in Tris buffered saline-Tween 20 for 2 h. Then, membranes were incubated with anti-osteocalcin (OCN) (1: 1000, Abcam, MA, USA), anti-osteopontin (OPN) (1: 1,000, Abcam, MA, USA), anti-RUNX2 (1: 1000, Abcam, MA, USA), anti-ERK1/2 (1: 1000, Abcam, MA, USA), anti-p-ERK1/2 (1: 1000, Abcam, MA, USA), anti-CRY2 (1: 1,000, Abcam, MA, USA), anti-Bax (1: 1,000, Abcam, MA, USA), anti-cleaved caspase3 (1: 500, Abcam, MA, USA), anti-Bcl2 (1: 2000, Abcam, MA, USA), anti-BMAL1 (1: 1000, Abcam, MA, USA), anti-AKT (1: 1000, Abcam, MA, USA), anti-p-AKT (1: 1000, Abcam, MA, USA), LC3B(1: 2000, Abcam, MA, USA) or anti-GAPDH (1: 2000, Abcam, MA, USA) overnight. Then, membranes were incubated with a secondary antibody conjugated with HRP (anti-rabbit IgG, 1: 5000, Cell Signaling Technology, Inc., MA, USA) for 1 h at room temperature. A Super Signal West Pico chemiluminescence detector (Thermo Fisher Scientific, Inc.) and Image Tool version 3.0 grayscale scanning software (Microsoft Corporation, Redmond, WA, USA) were used to observe and quantify protein bands in enhanced mode.

### Luciferase reporter assay

The partial sequence of the 3′-untranslated region (UTR) of CRY2 containing the putative binding sites of miR-27a-3p were synthetized and obtained from Sangon Biotech Co., Ltd. Subsequently, the sequences were cloned into the pmirGLO Dual-Luciferase miRNA Target Expression Vector (Promega Corporation) to construct wild-type or mutant (WT/MUT) reporter vectors for CRY2. Cells (2 × 10^4^) were seeded in 96-well plates and co-transfected with 100 ng PmirGLO-CRY2-WT or PmirGLO-CRY2-MUT and miR-27a-3p mimics with Lipofectamine 2000 reagent (Invitrogen). Luciferase activity was measured after 48 h of transfection.

### Cell apoptosis analysis

Cells were plated in 6-well plates (5 × 10^4^ per well). Cells were centrifuged at 956×*g* for 5 min and the residue was resuspended with 100 µL binding buffer. Subsequently, 5 µL Annexin V-FITC (Becton, Dickinson and Company, USA) and 5 µL propidium iodide (Becton, Dickinson and Company, USA) were added to the cells for 15 min at 4 °C. Apoptotic cells were analyzed by flow cytometry using a flow cytometer (Becton, Dickinson and Company, USA).

### Statistical analysis

Statistical analysis was performed using the SPSS 17.0 statistical software package (SPSS, Chicago, Illinois, USA). The data was expressed as the mean ± standard deviation (SD). One-way ANOVA (multiple groups) or Student’s t-test (two groups) was used to analyze differences between groups. All data represent the average of at least three independent experiments in triplicate, and P < 0.05 was considered to have significant difference.

## Results

### MiR-27a-3p and CRY2 are differentially expressed during osteoblast differentiation

**Fig. 1:**
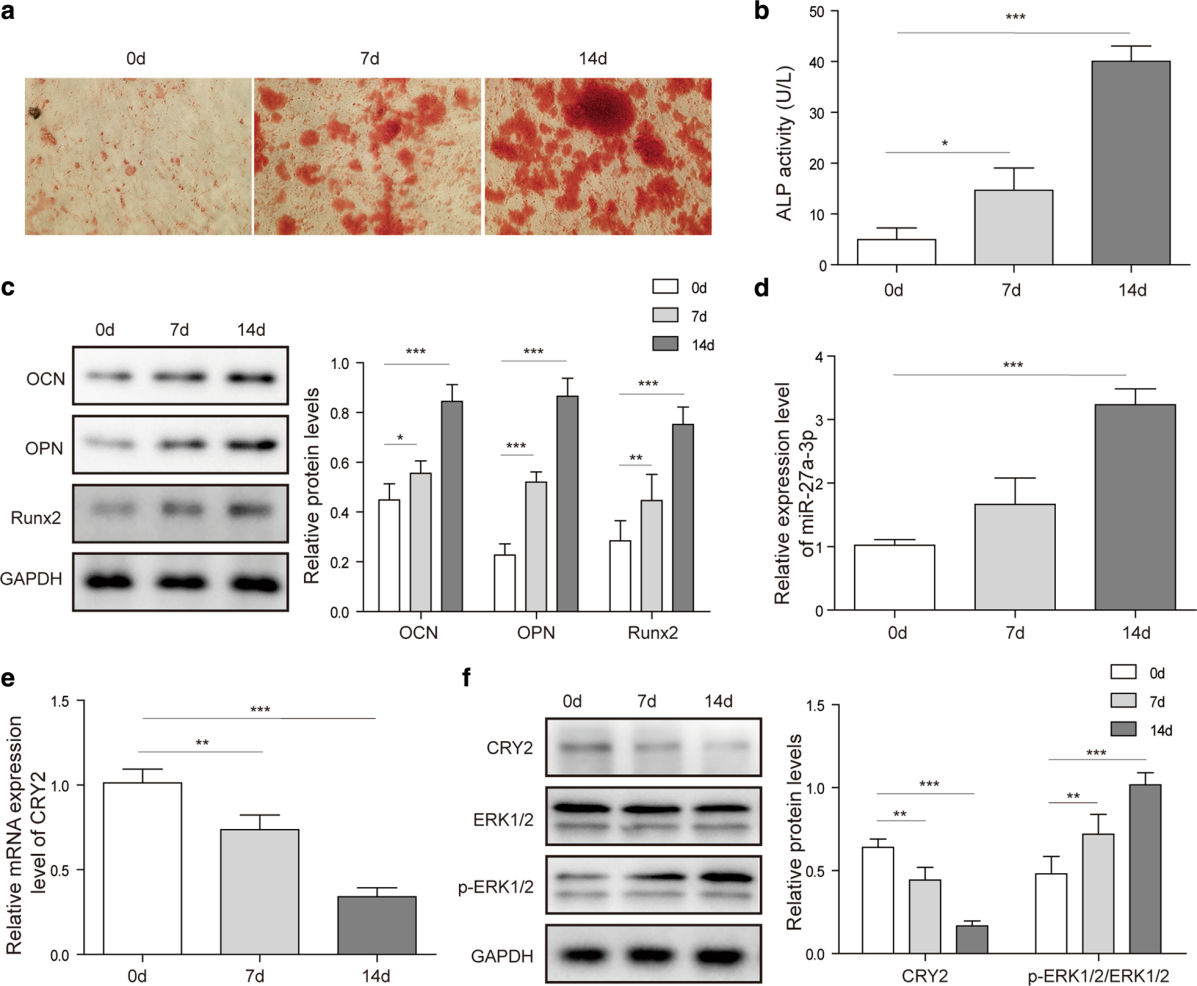
Expression of miR-27a-3p and CRY2 were detected in MC3T3-E1 cells. MC3T3-E1 cells were cultured with OIM. **a** Alizarin Red staining in MC3T3-E1 cells. **b** Detection of ALP activity. **c** The expression of OCN, OPN and RUNX2 were measured by western blotting. **d**, **e** RT-qPCR tested miR-27a-3p and CRY2 levels. **f** Western blotting analyzed the level of ERK1/2. *P < 0.05, **P < 0.01, ***P < 0.001

First, the expression of miR-27a-3p and CRY2 was investigated. The data revealed that osteogenic induction medium (OIM) increased the number of calcified nodules and ALP activity in cells in a time-dependent manner (Fig. 1a, b). In addition, OIM significantly increased the protein levels of OCN, OPN, and Runx2 (Fig. 1c). Interestingly, the expression of miR-27a-3p was markedly increased by OIM in a time-dependent manner (Fig. 1d), while the expression of CRY2 was decreased (Fig. 1e, f). Meanwhile, the phosphorylation level of ERK1/2 signaling pathway in MC3T3-E1 cells was activated by OIM in a time-dependent manner (Fig. 1f). All these results indicated that miR-27a-3p and p-ERK1/2 expression was upregulated in ODM-treated MC3T3-E1 cells, while CRY2 was inactivated.

### Knockdown of miR-27a-3p or overexpression of CRY2 inhibits osteoblast differentiation

**Fig. 2:**
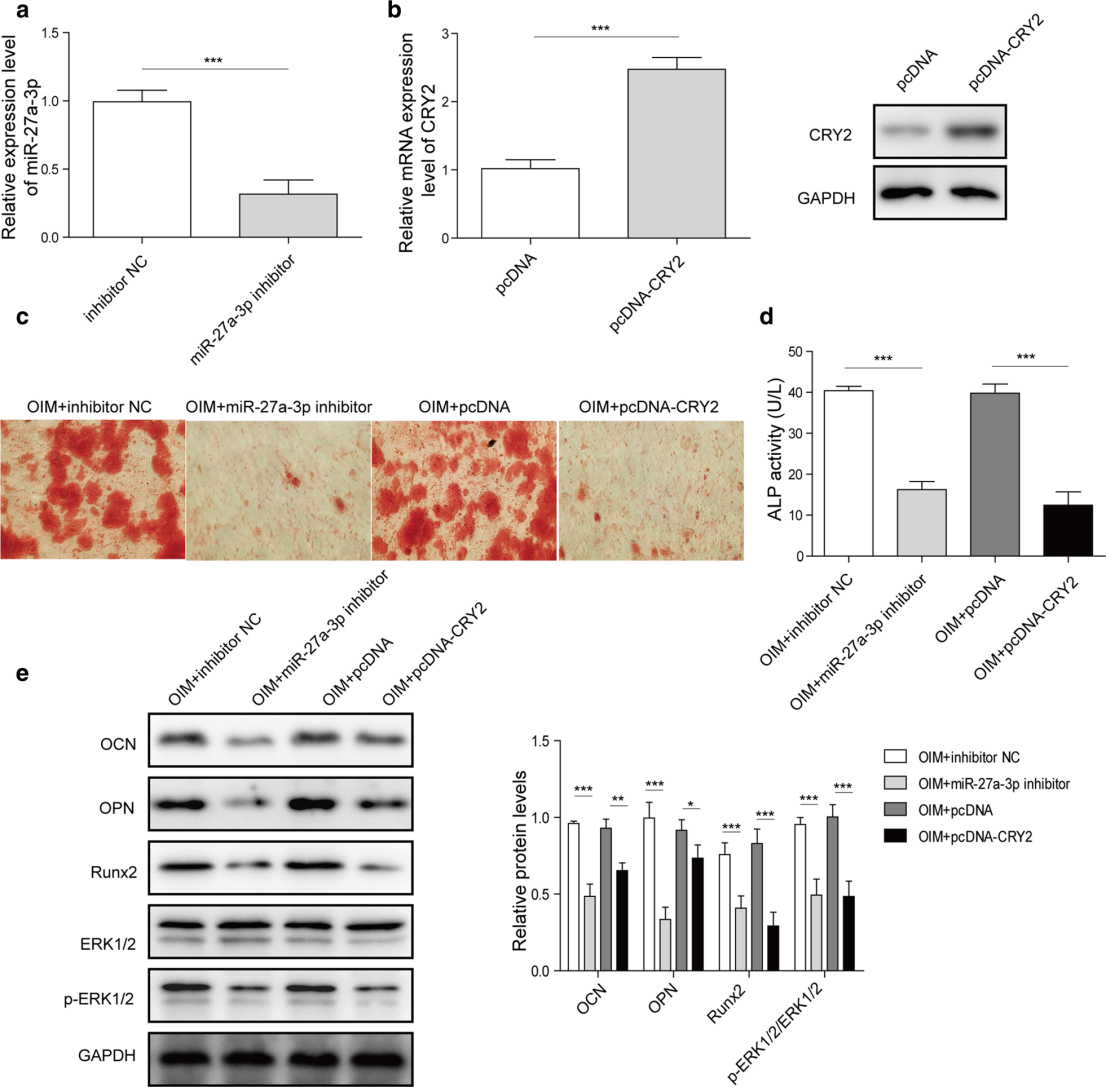
Knockdown of miR-27a-3p and overexpression of CRY2 inhibited ODM. MC3T3-E1 cells were transfected with miR-27a-3p inhibitor or CRY2 overexpression. **a** RT-qPCR detected the levels of miR-27a-3p. **b** RT-qPCR and western blotting examined CRY2 expression. **c** Alizarin Red staining in MC3T3-E1 cells. **d** Detection of ALP activity. **e** Western blotting analyzed the expression of OCN, OPN and RUNX2. *P < 0.05, **P < 0.01, ***P < 0.001

To investigate the functions of miR-27a-3p and CRY2 in osteoblast differentiation, MC3T3-E1 cells were transfected with CRY2 overexpression vector or miR-27a-3p inhibitor. The results showed that the miR-27a-3p expression was decreased in MC3T3-E1 cells when transfected with miR-27a-3p inhibitor (Fig. 2a). Increased expression of CRY2 mRNA and protein was observed in cells when transfected with pcDNA-CRY2 (Fig. 2b). In addition, the data of Alizarin red staining indicated that overexpression of CRY2 or miR-27a-3p inhibitor could reduce the osteoblastic mineralized nodules (Fig. 2c). Similarly, the ALP activity in MC3T3-E1 cells was notably decreased in the presence of miR-27a-3p downregulation or pcDNA-CRY2 (Fig. 2d). In summary, the expression of CRY2 interfered with the osteogenic differentiation of cells. In contrast, miR-27a-3p could enhance the osteogenic differentiation in MC3T3-E1 cells. In addition, the expressions of osteoblast differentiation-related proteins (OCN, OPN and Runx2) and p-ERK1/2 in MC3T3-E1 cells were notably decreased by CRY2 overexpression or miR-27a-3p inhibitor (Fig. 2e). Taken together, these results revealed that miR-27a-3p inhibitor or CRY2 overexpression could inhibit the osteoblast differentiation in MC3T3-E1 cells through inactivation of ERK1/2 signaling pathway.

### MiR-27a-3p activates ERK1/2 signaling by targeting CRY2

**Fig. 3:**
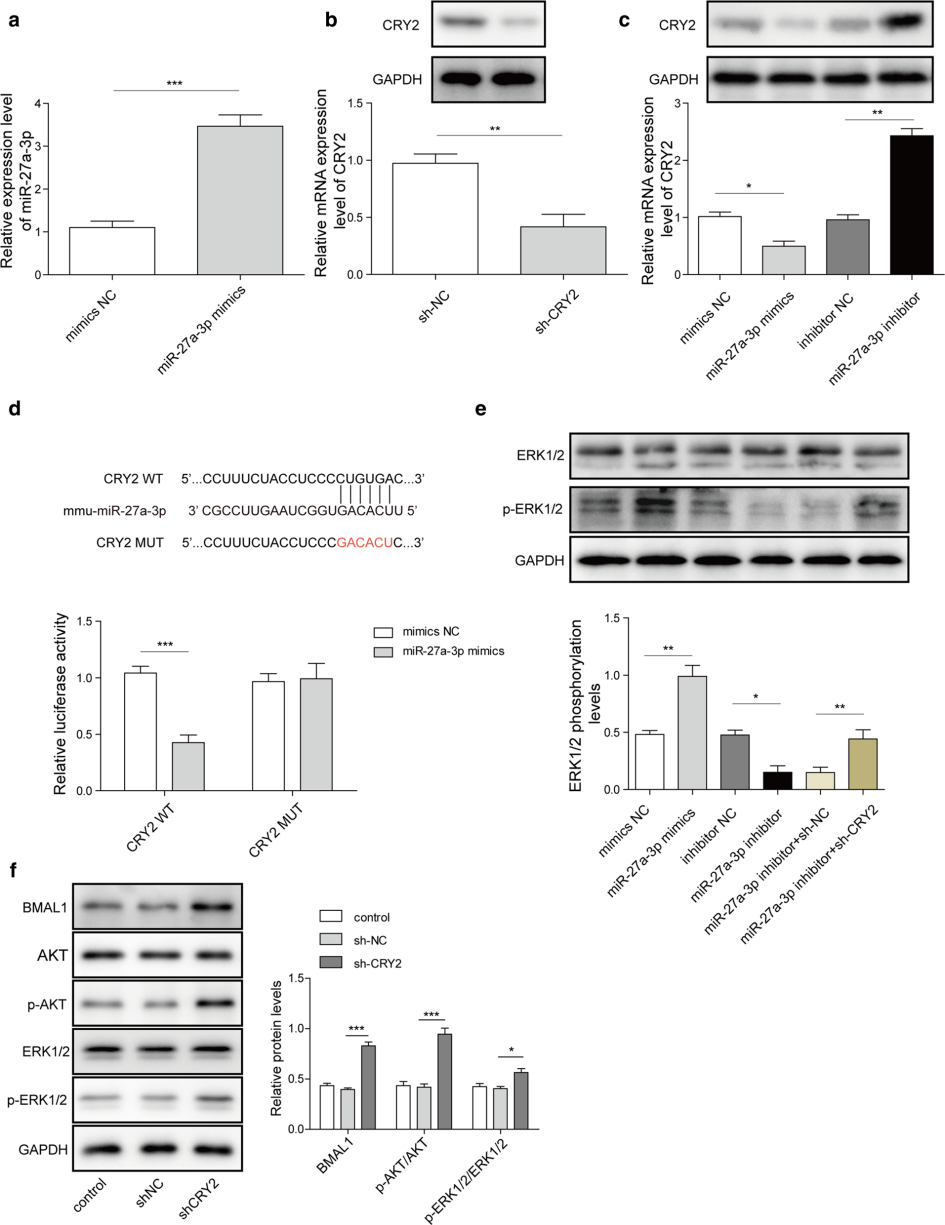
CRY2 was a direct target of miR-27a-3p. **a** RT-qPCR examined the expression of miR-27a-3p. **b**, **c** RT-qPCR and western blotting detected CRY2 expression. **d** Dual luciferase report analysis the interaction of miR-27a-3p and CRY2. **e** Western blotting analyzed ERK1/2 level. **f** MC3T3-E1 cells were transfected with CRY2 shRNA. The protein expressions of Akt, p-Akt, ERK, p-ERK and BMAL2 in MC3T3-E1 cells were detected by western blotting. *P < 0.05, **P < 0.01, ***P < 0.001

In Fig. 3a, c, the data of RT-qPCR showed that the level of miR-27a-3p in MC3T3-E1 cells was significantly increased when transfected with miR-27a-3p mimics, while the mRNA level of CRY2 was decreased (Fig. 3a, c). And the data of western blotting also showed a decrease in CRY2 expression (Fig. 3c). In addition, sh-CRY2 could notably reduce CRY2 expression in MC3T3-E1 cells (Fig. 3b). Meanwhile, bioinformatics software starbase (http://starbase.sysu.edu.cn/) predicted that CRY2 might be a direct target of miR-27a-3p (Fig. 3d), and the luciferase activity in WT-CRY2 was significantly inhibited when transfected with miR-27a-3p mimics, while miR-27a-3p had very limited effect on MUT-CRY2 (Fig. 3d). Additionally, overexpression of miR-27a-3p activated ERK1/2 signaling, while miR-27a-3p inhibitor exhibited the opposite effect. Meanwhile, miR-27a-3p inhibitor-induced inactivation of ERK1/2 signaling was reversed by CRY2 knockdown (Fig. 3e). Moreover, the expressions of BMAL1, p-Akt and p-ERK in MC3T3-E1 cells were significantly upregulated by knockdown of CRY2 (Fig. 3f). All these results suggested that miR-27a-3p could activate ERK1/2 pathway through binding with CRY2.

### MiR-27a-3p promotes osteoblast differentiation by downregulation of CRY2

**Fig. 4:**
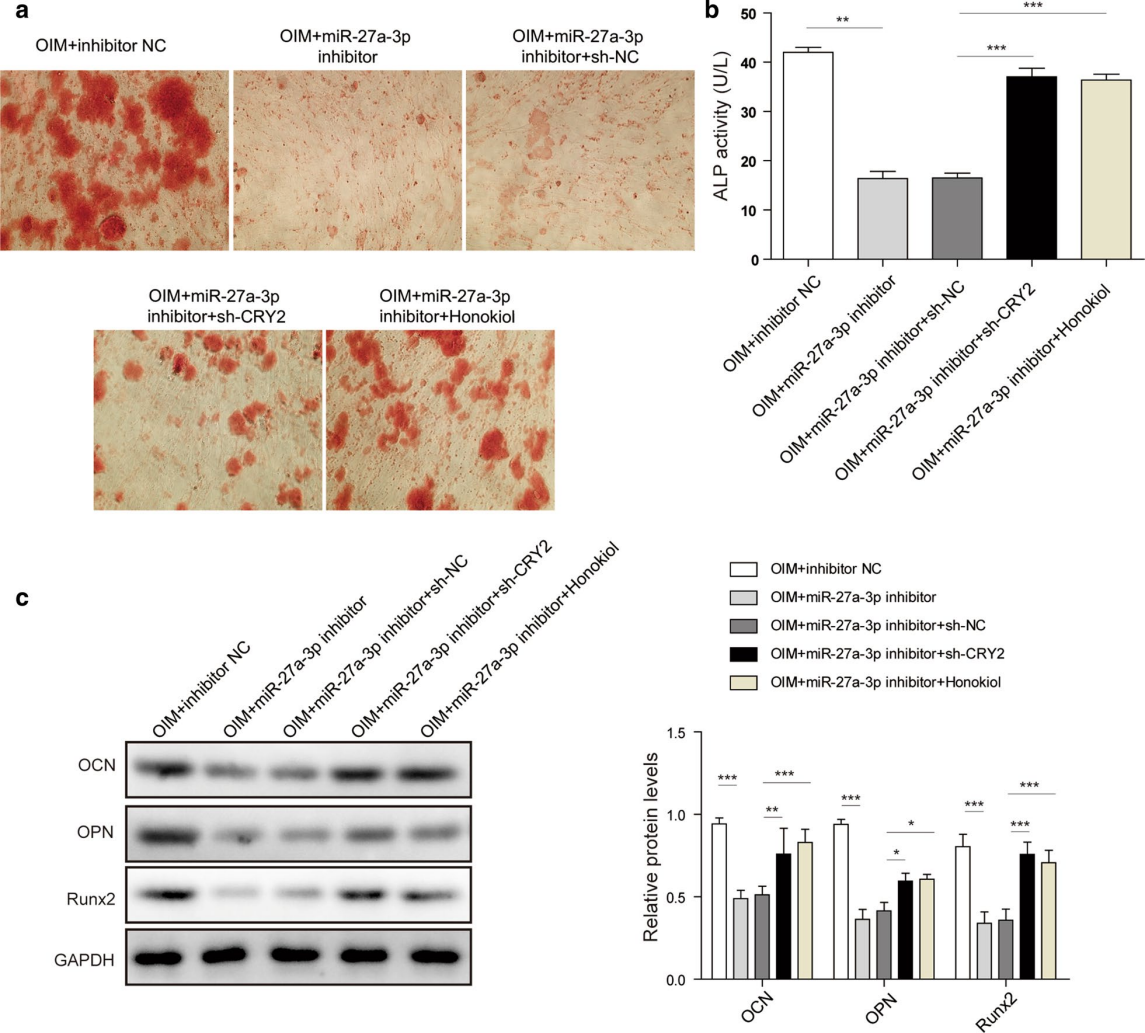
MiR-27a-3p regulated CRY2/ERK1/2 signal axis to promote ODM. MC3T3-E1 cells were treated with miR-27a-3p inhibitor or CRY2 shRNA. **a** Alizarin Red staining in MC3T3-E1 cells. **b** Detection of ALP activity; **c** Western blotting analyzed the expression of OCN, OPN and RUNX2. *P < 0.05, **P < 0.01, ***P < 0.001

Then, the mechanism by which miR-27a-3p regulated osteoblast differentiation was examined. The data revealed that miR-27a-3p inhibitor notably impeded the formation of cellular calcified nodules and ALP activity in osteoblasts,, while this phenomenon was rescued by knockdown of CRY2 or honokiol (ERK1/2 signal activator) (Fig. 4a, b). Meanwhile, the data of western blotting showed that miR-27a-3p inhibitor significantly reduced the levels of osteogenic proteins (Fig. 4c). Similarly, the effect of miR-27a-3p inhibitor was also offset by knockdown CRY2 or honokiol. Overall, miR-27a-3p promoted osteogenic differentiation in MC3T3-E1 cells through mediation of CRY2/ERK1/2 signaling pathway.

### miR-27a-3p inhibits cell apoptosis and induces autophagy via targeting CRY2

**Fig. 5:**
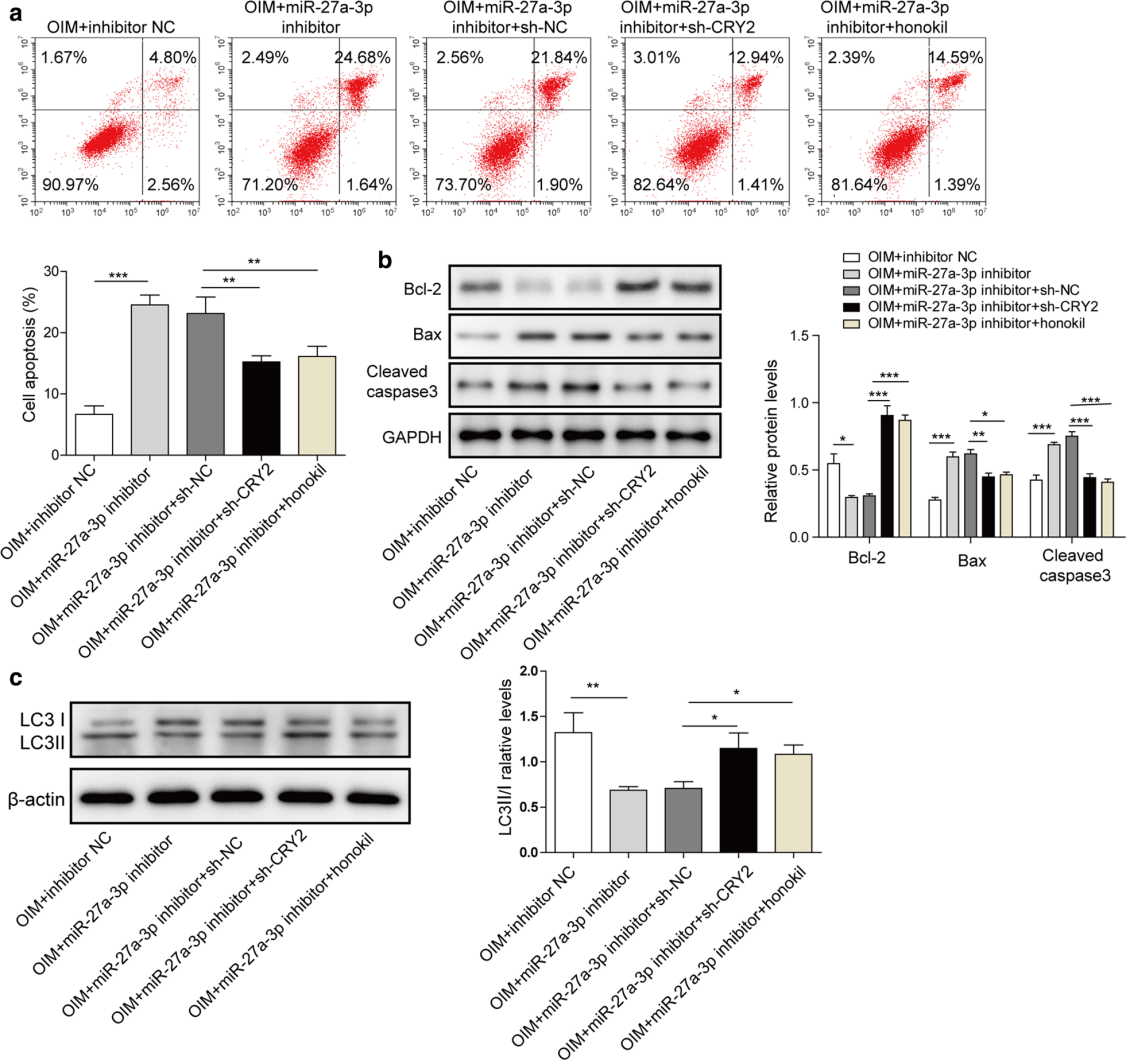
CRY2 knockdown could reverse the effect of miR-27a-3p inhibitor on cell apoptosis and autophagy. Cells were treated with miR-27a-3p inhibitor, miR-27a-3p inhibitor + CRY2 shRNA or miR-27a-3p inhibitor + honokiol. **a** The apoptosis of cells was tested by flow cytometry. **b** The protein expressions of Bax, Bcl-2 and cleaved caspase 3 were detected by western blotting. **c** The protein expressions of LC3 I and LC3 II were detected by western blotting. *P < 0.05, **P < 0.01, ***P < 0.001

In order to test the cell apoptosis, flow cytometry was performed. As revealed in Fig. 5a, knockdown of CRY2 or honokiol treatment significantly reversed miR-27a-3p inhibitor induced cell apoptosis. Meanwhile, knockdown of CRY2 inhibited the pro-apoptotic effect of miR-27a-3p inhibitor via mediation of Bax, Bcl-2 and cleaved caspase3 (Fig. 5b). In addition, knockdown of miR-27a-3p notably downregulated the ratio of LC3-II/LC3-I, however, CRY2 shRNA or honokiol treatment reversed this phenomenon (Fig. 5c). Altogether, CRY2 knockdown or honokiol reversed the effect of miR-27a-3p inhibitor on cell apoptosis and autophagy.

## Discussion

Nowadays, the major treatment for osteoporosis was to slow the absorption of bones or induce the formation of bones (Rachner et al. 2011). Osteoblasts were necessary for bone development and formation, and accelerating osteoblast differentiation was a promising method to promote bone formation (Wang and Cai 2020). In this study, we firstly explored the function by which miR-27a-3p promoted the osteogenic differentiation. As expected, CRY2 was identified to be the target of miR-27a-3p, and miR-27a-3p regulated CRY2/ERK1/2 signaling to induce osteoblast differentiation. In addition, our study firstly explored the relation among miR-27a-3p, CRY2 and ERK1/2, suggesting that miR-27a-3p/CRY2/ERK1/2 could play an important role in progression of osteoporosis.

Dysregulation of miRNAs are closely involved in many diseases, including the imbalance between adipogenic differentiation and osteogenic differentiation, thus leading to the occurrence of osteoporosis (Ha 2011; Nugent 2016). Among them, highly expressed miR-27a might be closely correlated with the development of osteoporosis (Gu et al. 2016; Guo et al. 2014). Previous reports indicated that miR-27a downregulation could induce adipogenic differentiation, while miR-27a suppressed fat formation and aggrandized osteogenesis by regulating PPARγ and GREM1 (Gu et al. 2016). In this study, miR-27a-3p expression was upregulated in OIM-induced MC3T3-E1 cells. MiR-27a-3p knockdown impeded the osteogenic differentiation and inhibited the phosphorylation of ERK1/2. Our study was consistent with the previous research about the role of ERK in osteogenic differentiation (Kim et al. 2019; Sun et al. 2018). In addition, this study further revealed that miR-27a-3p was involved in osteogenic differentiation and ERK pathway activation. Meanwhile, previous data showed that miR-27a-3p knockdown could inhibit the phosphorylation of ERK1/2 (Su et al. 2019; Zhou et al. 2016). Thus, our study further confirmed that miR-27a-3p could act as a promoter of ERK.

Starbase prediction showed that miR-27a-3p targeted binding sites existed on CRY2. CRY2 is a blocker protein that negatively regulates target gene transcription and participates in regulating various physiological processes in the body (Lee et al. 2010). In addition, disruption of the circadian rhythm might cause specific bone formation mechanisms to be blocked, implying that CRY2, as one of the main molecules of circadian rhythm, might participate in the bone formation (Vriend and Reiter 2016; Zhou et al. 2018). In particular, the CRY2 participates in the synthesis and catabolism of bones, while its mechanism is still unclear (Maronde et al. 2010). Initially, CRY2 expression in MC3T3-E1 cells was markedly weakened during osteogenic differentiation. However, overexpression of CRY2 inhibited the ODM *in vitro*, indicating that CRY2 negatively regulated osteogenic differentiation activities. Meanwhile, our findings indicated that CRY2 knockdown could reverse the effect of miR-27a-3p inhibitor on osteogenic differentiation. Tsuchiya Y et al. found that CRY2 knockdown could lead to the loss of p-ERK (Tsuchiya et al. 2013). Our study was consistent to this previous research. On the other hand, a previous reference indicated that CRY2 knockdown increased the phosphorylation of ERK 1/2 in cancer cells (Yu et al. 2018). In addition, knockdown of SPRY4 promoted osteogenic differentiation by enhancing MAPK mediated ERK phosphorylation (Park et al. 2019). Our current study found that ERK pathway activation and CRY2 knockdown reversed the regulation of miR-27a-3p inhibitor on osteoblasts differentiation, suggesting inhibition of miR-27a-3p reduced activation of p-ERK was reversed by CRY2 knockdown. Based on the previous references and our data, miR-27a-3p could activate ERK signaling through regulation of CRY2.

It had confirmed that autophagy played an important role in osteoblast differentiation (Li et al. 2019). Despite their distinct mechanisms and functions, apoptosis and autophagy were closely associated. Importantly, autophagy was necessary for the onset of apoptosis, which usually triggers the occurrence of apoptosis (Wang et al. 2019). A study showed that miR-27a-3p inhibitor suppressed the apoptosis of osteoblasts via inducing autophagy. ERK was known to be a mediator in cell proliferation through inducing the cell autophagy (Cagnol and Chambard 2010; Zhang et al. 2019). Consistent with this view, our data revealed that miR-27a-3p could mediate CRY2/ERK1/2 in osteoblasts and downregulation of miR-27a-3p induced apoptosis in osteoblasts via mediation of CRY2/ERK1/2. Dong Y et al. found that miR-27a-3p could regulate the autophagy in LPS-treated HUVECs through targeting SLIT2 (Dong et al. 2020; Xie et al. 2020). LC3 was considered to play regulatory roles in autophagy (Wang et al. 2020). In present study, (Zheng et al. 2020; Zhou et al. 2020) we found that CRY2 knockdown or honokil reversed the effect of miR-27a-3p inhibitor on osteogenic differentiation via regulating apoptosis and autophagy. Furthermore, co-transfection of (Wang et al. 2020) miR-27a-3p inhibitor and CRY2 shRNA or honokil induced cell autophagy via mediation of LC3.

Of course, there are some limitations in this study as follows: (1) more target mRNAs of miR-27a-3p need to be explored; (2) the association between miR-27a-3p and CRY2/ERK needs to be further confirmed in vivo. Thereby, more investigations are needed in future. In conclusion, we manifested that miR-27a-3p enhanced OPN, OCN and RUNX2 gene expression by regulating the CRY2/ERK1/2 axis in MC3T3-E1 cells to promote osteogenic differentiation. These findings in this study indicated that miR-27a-3p was the regulatory factor of ODM and a potential therapeutic target to promote osteoblast differentiation against osteoporosis.

## Data Availability

All data generated or analyzed during this study are included in this published article.
